# Thermosonication improves the bioactive properties and *in vitro* bioaccessibility of broccoli juice through integrated RSM and metaheuristic optimization

**DOI:** 10.3389/fnut.2026.1880853

**Published:** 2026-06-25

**Authors:** Tuba Eda Arpa Zemzemoğlu, Nazan Tokatlı Demirok, Melikenur Türkol, Remzi Gürfidan, Oğuzhan Kilim, Nazlı Tokatlı, Seydi Yikmiş, Isam A. Mohamed Ahmed, Moneera O. Aljobair, Abdullah Yinanç

**Affiliations:** 1 Nutrition and Dietetics, Faculty of Health Science, Gümüşhane University, Gümüşhane, Türkiye; 2 Nutrition and Dietetics, Faculty of Health Sciences, Tekirdağ Namık Kemal University, Tekirdağ, Türkiye; 3 Isparta Vocational School of Information Technologies, Database, Network Design and Management, Isparta University of Applied Science, Isparta, Türkiye; 4 Department of Computer Engineering, Faculty of Engineering and Natural Sciences, Istanbul Health and Technology University, Istanbul, Türkiye; 5 Department of Food Technology, Tekirdağ Namık Kemal University, Tekirdağ, Türkiye; 6 Department of Food Sciences and Nutrition, College of Food and Agricultural Sciences, King Saud University, Riyadh, Saudi Arabia; 7 Department of Sports Health, College of Sports Sciences and Physical Activity, Princess Nourah Bint Abdulrahman University, Riyadh, Saudi Arabia; 8 Vocational School of Technical Sciences, Tekirdağ Namık Kemal University, Tekirdağ, Türkiye

**Keywords:** bioactive compounds, broccoli juice, *in vitro* digestion, metaheuristic optimization, thermosonication

## Abstract

Broccoli juice is highly nutritious, containing phenolic compounds, chlorophyll derivatives, antioxidants, and other biologically active phytochemicals; however, conventional thermal processing may reduce their stability and bioaccessibility. The aim of this study was to analyse the impact of thermosonication on the following properties of broccoli juice: bioactivity, antioxidant capacity, phenolic profile and *in vitro* bioaccessibility. Thermosonication conditions were optimized using Response Surface Methodology (RSM) integrated with the Gray Wolf Optimizer (GWO) and Particle Swarm Optimization (PSO) algorithms. Broccoli juice samples were subjected to conventional pasteurization and thermosonication under different processing conditions, and total chlorophyll, ascorbic acid (AA), ferric reducing antioxidant power (FRAP), total phenolic content (TPC), and individual phenolic compounds were evaluated. Digestive stability and post-digestion bioaccessibility were also assessed using a standardized *in vitro* gastrointestinal digestion model. The developed quadratic models exhibited high statistical significance for TPC and FRAP responses (*p* < 0.001), with coefficients of determination (*R*^2^) of 0.9949 and 0.9853, respectively. The optimum thermosonication conditions were identified as 40 °C, 6.75 min, and 64.04% amplitude, under which experimentally validated FRAP and TPC values reached 12.86 mmol TE/L and 149.77 mg GAE/100 ml, respectively. HPLC-DAD analysis showed that gallic acid and naringin were the pre-dominant phenolic compounds, and that thermosonication promoted greater phenolic retention than conventional pasteurization. *In vitro* digestion results demonstrated that thermosonicated broccoli juice maintained significantly higher levels of TPC, FRAP, chlorophyll, and AA throughout gastrointestinal digestion, with higher TPC recovery (30.45%) than pasteurized samples (26.22%), indicating improved post-digestion bioaccessibility of phenolic compounds. Similar improvements were observed for antioxidant capacity and chlorophyll retention. Furthermore, PSO and GWO optimization results showed strong agreement with RSM predictions, confirming the robustness of the proposed optimization strategy. Overall, thermosonication was shown to effectively and sustainably enhance the functional quality, antioxidant properties, phenolic stability, and digestive bioaccessibility of broccoli juice.

## 1 Introduction

Broccoli (*Brassica oleracea* var. italica Plenck) is a popular food (1). The term ‘broccoli' is used to describe the young, edible stems and flower stalks of the plant (2). Broccoli, belonging to the *Brassicaceae* family, is notable for its rich phytochemical content and various health benefits (3). Broccoli is a cruciferous vegetable that contains a variety of nutrients, including carotenoids such as neoxanthin, lutein and violaxanthin, as well as β-carotene, and minerals such as potassium, selenium, zinc, iron, phosphorus, calcium, and magnesium (4, 5). Broccoli florets contain high levels of glucosinolates (GLS), including glucoerucin (GLE) and glucoraphanin (GLR) (6). Glucosinolates and their degradation products have beneficial properties, including anticancer, antimicrobial, metabolic syndrome-regulating, antioxidant, anti-inflammatory, renoprotective, and neuroprotective effects (7, 8). Sulforaphane, in particular, is being studied for its regulation of redox-sensitive pathways and cellular detoxification enzymes; indole-3-carbinol also plays an important role in regulating DNA repair systems and estrogen metabolism (9).

The rich nutrients and bioactive components of broccoli can also be utilized by consuming broccoli juice. However, traditional preservation methods involving high-temperature treatment can degrade heat-sensitive bioactive compounds, particularly sulforaphane, and reduce their functional properties, despite extending the product's shelf life (10). Due to the negative effects of thermal processes, there has been a growing trend in the literature toward non-thermal technologies that have demonstrated high success in preserving quality (11–13). At the same time, interest in using combined systems to further increase the efficiency of these technologies has accelerated (14, 15).

Thermosonication is a method that combines heat and ultrasonication (16). Thermosonic processing shows promise in both improving juice quality and enhancing its safety characteristics (17). Thermosonication can bring about various physical and mechanical changes in liquid food systems, such as juice (18). Therefore, mathematical modeling is essential for improving understanding of, and optimizing the effectiveness of, thermosonication on juice quality.

Given the rich bioactive composition of broccoli, the effect of thermosonication on these components is critical for functional food technologies. This study aims to make a detailed comparison of the effects that different ways of processing broccoli juice have on its physicochemical and bioactive properties. In this context, changes in total TPC, FRAP, total chlorophyll, ascorbic acid, and individual phenolic compounds in the unprocessed control group (CON-B), the conventional pasteurization group (PAS-B), and the thermosonication group (TS-B), an innovative processing technology, were systematically investigated. In addition, the digestive stability and *in vitro* bioaccessibility of these bioactive compounds were evaluated using a standardized gastrointestinal digestion model. The originality of this study is based on the combined application of Response Surface Methodology (RSM) and metaheuristic optimization algorithms, including Gray Wolf Optimizer (GWO) and Particle Swarm Optimization (PSO), for the optimization of thermosonication conditions in broccoli juice, together with a comprehensive investigation of bioactive properties, phenolic profile, antioxidant activity, and post-digestion bioaccessibility. Taking a multidisciplinary approach, our study aims to contribute a comprehensive dataset to the effective use of thermosonication to improve the quality of broccoli juice, both theoretically and experimentally.

Although many research studies have explored the impact of thermosonication on the quality features of fruit and vegetable juices, information on optimizing thermosonication processing conditions for broccoli juice remains limited. Furthermore, studies evaluating the digestive stability and *in vitro* bioaccessibility of bioactive compounds in thermosonicated broccoli juice are scarce. To the best of our knowledge, no previous study has combined RSM with metaheuristic optimization approaches, such as PSO and GWO, while simultaneously evaluating the post-digestion bioaccessibility of bioactive compounds in broccoli juice. Therefore, the present study aimed to optimize thermosonication conditions and investigate their effects on antioxidant properties, phenolic profile, digestive stability, and *in vitro* bioaccessibility of broccoli juice.

## 2 Materials and methods

### 2.1 Preparation of broccoli juice

The fresh broccoli (*Brassica oleracea var*. italica) samples used in this study were purchased from a local market in Tekirdag, Türkiye. Prior to juice production, the broccoli florets were carefully washed to remove any foreign matter. The samples were then chopped with a knife and homogenized using a Waring Commercial Blender (Model HGB2WTS3, St. Louis, MO, USA) to obtain broccoli juice. Unprocessed fresh broccoli juice samples were considered the control group and coded as CON-B. The prepared samples were stored at 4 °C until analysis.

### 2.2 Thermal pasteurization

The pasteurization of the broccoli juice involved transferring the samples into 100-ml glass bottles and heating at 85 ± 1 °C for 2 min in a Wisd model WUC-D06H water bath (Daihan, Korea). This condition was selected based on previous studies on fruit and vegetable juices, in which temperatures around 85 °C have been widely used to ensure microbial safety while minimizing degradation of quality attributes and bioactive compounds. Furthermore, this treatment served as a benchmark for conventional thermal processing, enabling a direct comparison with thermosonication. Following thermal treatment, all broccoli juice samples were rapidly cooled to room temperature in an ice-water bath. The pasteurized broccoli juice samples were designated as pasteurized broccoli juice (PAS-B) and stored at −18 ± 1 °C until further analysis.

### 2.3 Thermosonication procedure

Samples of broccoli juice (100 ml) were treated with thermosonication in an ultrasonic processor (Hielscher Ultrasonics UP200St, Germany). It operates at 200 W and 26 kHz and is equipped with a 14-mm-diameter titanium probe (sonotrode). The experimental variables examined during the treatment included processing temperature (*X*1, 40–60 °C), time (*X*_2_, 6–12 min), and ultrasound amplitude (*X*3, 60%−100% amp). All applications were performed under continuous operating conditions. To maintain temperature stability and prevent undesirable thermal effects during sonication, the sample containers were immersed in an ice-water bath throughout the treatment. Following thermosonication, the samples were preserved at −18 ± 1 °C prior to subsequent analyses. The treated samples were designated as thermosonicated broccoli juice (TS-B).

### 2.4 Response surface methodology (RSM)

This study evaluated and optimized the effects of thermosonication processing parameters on FRAP and TPC using RSM. The independent variables selected for the experimental design were processing temperature (*X*1, °C), treatment time (*X*_2_, min), and ultrasound amplitude (*X*3, %). To improve naive error estimation and ensure reliable discrepancy analysis, a total of 20 experimental trials were conducted, including five replicates at the center point (*X*1 = 50 °C, *X*_2_ = 8 min, and *X*3 = 80%). A second-order (quadratic) polynomial model was used to fit the experimental data and describe the association among the response factors and processing parameters (Equation 1).


$$\begin{array}{l} Y=\;\beta_{0}+\sum \beta_{i}X_{i}+\;\sum \;\beta_{ii}{X_{I}}^{2}+\sum \beta_{ij}X_{i}X_{j} \end{array}$$ (1)

### 2.5 Determination of bioactive substances and antioxidant activity

The antioxidant activity assay was performed to determine the capacity of the broccoli juice samples to reduce ferric ions (Fe3?) to ferrous ions (Fe^2^?), thereby measuring their antioxidant capacity. The FRAP reagent was freshly made by combining acetate buffer solution (300 mM, pH 3.6), a solution of TPTZ (10 mM) dissolved in HCl (40 mM), and a 20 mM solution of FeCl3·6H_2_O in a volume ratio of 10:1:1 (v/v/v). Prior to analysis, the reagent mixture was incubated at 37 °C for 10 min. Absorbance readings were recorded at 593 nm, and antioxidant capacity values were quantified using a Trolox calibration curve. The findings were presented as mmol Trolox equivalent per liter (mmol TE/L) (19). To measure the TPC, the Folin–Ciocalteu reagent (Sigma-Aldrich, F9252) was added undiluted to measure the TPC. A freshly prepared sodium carbonate solution (7.5%) was made by dissolution of Na_2_CO3 (Merck, ≥99%). After adding the reagents, the reaction mixtures were incubated at room temperature in the dark for 30 min prior to analysis (20).

The ascorbic acid content of the broccoli juice samples was analyzed via a titrimetric method, which is based on the principle of reducing 2,6-dichlorophenolindophenol (DCPIP). Prior to analysis, 0.2 g of oxalic acid was added to 30 ml of broccoli juice to stabilize the vitamin C. Then, 10 ml of the prepared mixture was titrated with a DCPIP solution until a persistent light-pinkcolor was obtained. Ascorbic acid levels were calculated and are given in milligrams per 100 ml (21).

Chlorophyll was determined using a spectrophotometric procedure. Broccoli juice samples were mixed with 80% acetone to transfer plant pigments into the solvent. The solution was clarified through repeated filtration with Whatman filter paper. The optical density values of the filtrate were recorded at 645 and 663 nm to determine the chlorophyll amount (22).

### 2.6 Characterization of individual phenolic compounds by HPLC-DAD analysis

**Figure 1:**
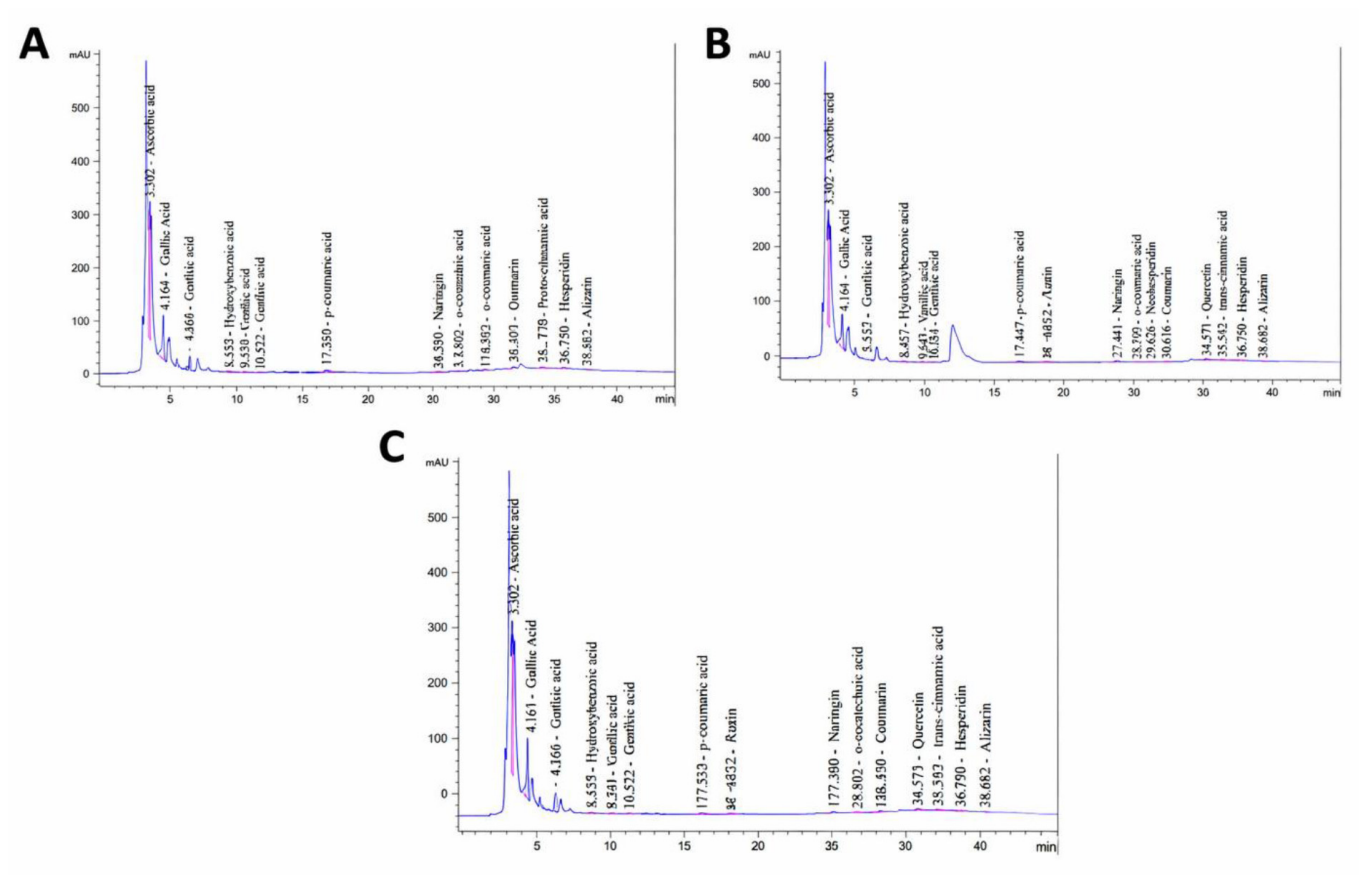
Representative HPLC–DAD chromatograms of broccoli juice phenolic profile for **(A)** control untreated sample (CON-B), **(B)** pasteurized sample (PAS-B), and **(C)** thermosonicated sample (TS-B). Peaks were assigned by matching retention times and UV–Vis spectra with authentic standards analyzed under identical conditions; chromatograms illustrate the main identified compounds, including gallic acid, quercetin, naringin, protocatechuic acid, hydroxybenzoic acid, gentisic acid, vanillic acid, *p*-coumaric acid, rutin, neohesperidin, coumarin, trans-cinnamic acid, hesperidin, and alizarin.

Individual phenolic compounds in broccoli juice were analyzed in the control (CON-B; untreated), pasteurized (PAS-B), and thermosonicated (TS-B) samples. Prior to HPLC injection, samples were clarified to remove suspended solids (e.g., by centrifugation) and filtered through a membrane syringe filter. Chromatographic analysis was carried out with an Agilent 1260 HPLC system fitted to an ACE Genix C18 column and a diode-array detector (DAD). The column temperature was set to 30 °C, the flow rate to 0.80 ml/min and the injection volume to 10 μl. The mobile phase consisted of water containing 0.1% (v/v) phosphoric acid and an organic phase. A gradient elution program was used to perform the separation under conditions previously optimized (23, 24). DAD detection was performed at 360, 320, and 280 nm. The phenolic substances were determined by analyzing the UV-Vis spectra and retention times of the broccoli juice samples alongside authentic standards, all examined under identical conditions. Quantitative analyses were completed based on external calibration curves generated with standards ranging from gallic acid to alizarin (Figure 1).

### 2.7 Application of simulated gastrointestinal digestion *in vitro*

The gastrointestinal digestion process of broccoli juice samples was investigated using a static *in vitro* digestion model based on the standardized INFOGEST method (25). Briefly, oral digestion was conducted by mixing broccoli juice with simulated salivary fluid (SSF) containing α-amylase (75 U/ml final activity), followed by incubation at 37 °C for 2 min. Gastric digestion was initiated by adding simulated gastric fluid (SGF) containing pepsin (2,000 U/ml final activity), adjusting the pH to 3.0, and incubating the samples at 37 °C for 2 h under continuous shaking. Subsequently, intestinal digestion was performed by adding simulated intestinal fluid (SIF) containing bile salts (10 mM final concentration) and pancreatin (100 U/ml trypsin activity). The pH was adjusted to 7.0, and broccoli juice samples were incubated at 37 °C for an additional 2 h under continuous agitation.

### 2.8 Statistical analysis

The statistical analyses were conducted using SPSS 22.0 software (SPSS Inc., Chicago, IL, USA). All analyses were repeated three times, and data were expressed as mean ± SD. Statistical differences between groups were evaluated using ANOVA and Tukey's HSD test (*p* < 0.05).

## 3 Results and discussion

### 3.1 Effects of different processing conditions on the antioxidant activity and bioactive compounds

**Figure 2:**
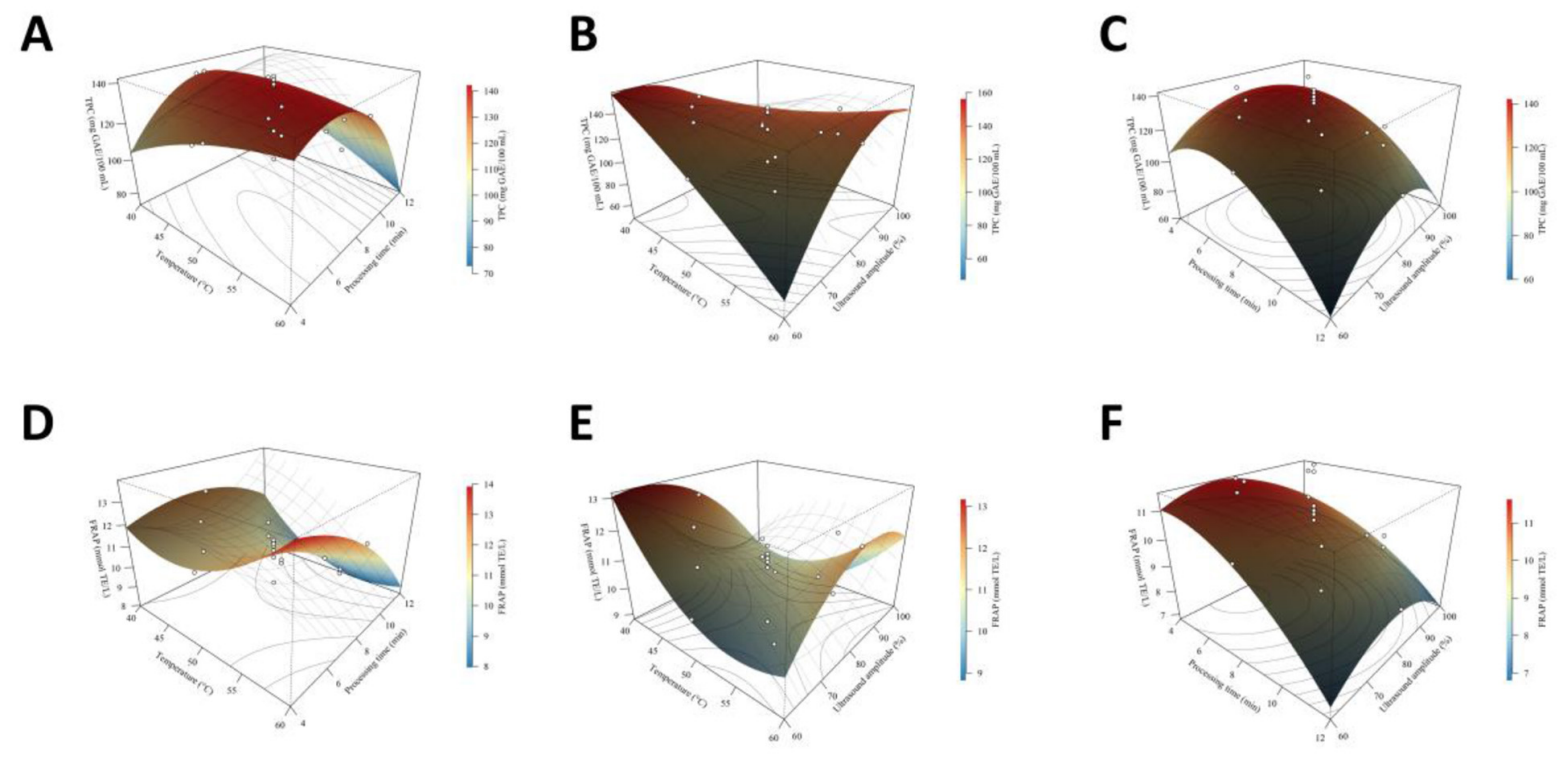
Three-dimensional response surfaces and their corresponding contour plots demonstrate the interactive effects of thermosonication parameters on broccoli extract responses **(A–C)**, total phenolic content (TPC), and **(D–F)** ferric reducing antioxidant capacity (FRAP). The graphs present the interactions between temperature and treatment time, temperature and ultrasound amplitude, and treatment time and ultrasound amplitude, respectively, with the other variable held at a constant medium level. Additionally, experimental data were overlaid on the corresponding surface plots to visually demonstrate the model's fit.

The three-dimensional response surface plots presented in Figure 2 demonstrate that thermosonication parameters exerted non-linear and distinctly quadratic effects on both TPC and FRAP values (Figures 2A–F). For TPC (Figures 2A–C), the presence of peak/plateau regions on the response surfaces and the concentration of contour lines at the base of the plots indicate that the response was not governed by a simple linear increase or decrease associated with a single factor. Instead, the variation in TPC became more pronounced due to the combined effects of two processing variables. The overall agreement between the experimental data points and the predicted surfaces, together with the close distribution of center point replicates, suggests that the response remained relatively stable within the intermediate processing region and that the developed model adequately represented the observed variations in this range (Table 1). Similarly, the FRAP response surfaces (Figures 2D–F) exhibited sharper slope transitions in certain factor planes, indicating that the antioxidant capacity of broccoli juice was highly sensitive to changes in thermosonication processing conditions.

**Table 1:** RSM-predicted responses, including the optimum (fit) and confirmatory replicate (uncoded units).

Entry	*X*1 (temp, °C)	*X*_2_ (time, min)	*X*3 (amp, %)	TPC exp (mg GAE/100 ml)	TPC Pred	FRAP exp (mmol TE/L)	FRAP pred
1	45	6.00	70.00	143.91	143.50	12.04	11.92
2	50	8.00	80.00	137.30	139.97	10.56	10.86
3	45	6.00	90.00	107.84	108.97	10.65	10.56
4	45	10.00	90.00	105.95	106.15	9.89	9.79
5	50	12.00	80.00	95.94	95.28	8.43	8.32
6	50	8.00	60.00	113.34	113.21	10.23	10.12
7	55	6.00	70.00	126.71	127.88	11.57	11.69
8	55	10.00	70.00	101.14	101.38	9.24	9.33
9	55	6.00	90.00	139.21	138.97	11.70	11.59
10	50	8.00	80.00	140.38	139.97	10.95	10.86
11	50	8.00	80.00	139.94	139.97	10.95	10.86
12	55	10.00	90.00	118.17	119.97	9.45	9.58
13	50	4.00	80.00	124.10	124.59	11.34	11.45
14	50	8.00	80.00	141.71	139.97	11.06	10.86
15	50	8.00	100.00	97.31	97.27	8.90	9.00
16	40	8.00	80.00	135.83	135.76	12.44	12.54
17	60	8.00	80.00	134.06	133.96	12.22	12.10
18	50	8.00	80.00	137.74	139.97	10.77	10.86
19	50	8.00	80.00	141.71	139.97	10.90	10.86
20	45	10.00	70.00	131.56	133.18	10.67	10.80
OPT (fit)	40	6.75	64.04		154.63		13.25
OPT (exp rep.)	40	6.75	64.04	149.77		12.86	

X1, temperature (°C); X_2_, processing time (min); X3, ultrasound amplitude (%); FRAP, ferric reducing antioxidant power; TPC, total phenolic content; TE, Trolox equivalents; GAE, gallic acid equivalents; Exp, experimental; Pred, predicted; RSM, response surface methodology.

The statistical validity of the developed models was further confirmed by ANOVA and goodness-of-fit parameters. The quadratic models constructed for both response variables were statistically highly significant (*p* = 0.000). Low residual error values and an insignificant discordance test result indicated that the experimental data were successfully represented by the quadratic model and that the model fit the dataset well (Table 2).

**Table 2:** Compact ANOVA summary and model fit statistics for the quadratic models (FRAP and TPC).

Response	Source	DF	Adj SS	Adj MS	*F*-value	*P*-value
FRAP	Model	9	22.2416	2.47129	74.36	0.000
	Linear	3	11.2503	3.75010	112.84	0.000
	Square	3	9.3847	3.12824	94.13	0.000
	2-way interaction	3	1.6066	0.53553	16.11	0.000
	Error	10	0.3323	0.03323		
	Lack-of-fit	5	0.1761	0.03521	1.13	0.450
	Pure error	5	0.1563	0.03126		
	Total	19	22.5740			
TPC	Model	9	5,122.5500	569.17000	218.56	0.000
	Linear	3	1,119.2400	373.08000	143.26	0.000
	Square	3	2,804.0100	934.67000	358.91	0.000
	2-way interaction	3	1,199.3000	399.77000	153.51	0.000
	Error	10	26.0400	2.60000		
	Lack-of-fit	5	7.8900	1.58000	0.43	0.809
	Pure error	5	18.1500	3.63000		
	Total	19	5,148.5900			
Model fit statistics						
**Response**	**S**	***R*** ^2^	***R***^2^ **(adj)**	***R***^2^ **(pred)**		
FRAP	0.182302	0.9853	0.9720	0.9271		
TPC	1.613760	0.9949	0.9904	0.9836		

MS, adjusted mean square; Adj SS, adjusted sum of squares; DF, degrees of freedom; Adj F, F-statistic; LoF, lack of fit. Model fit: S, standard error of regression; R^2^, coefficient of determination; R^2^(pred), predicted R^2^; R^2^(adj), adjusted R^2^.

The goodness-of-fit statistics also supported these findings. The high determination coefficients obtained for FRAP (*R*^2^ = 0.9853) and TPC (*R*^2^ = 0.9949) demonstrated that a substantial proportion of the variability in the responses was successfully explained by the developed models. These results suggest that the peak and valley regions visually observed in the response surface plots were supported by a statistically reliable modeling framework (Table 2). Therefore, the response surfaces shown in Figure 2 should be considered not only graphical representations but also robust model outputs that support optimization decisions.

A strong agreement was observed between the experimentally validated results and the optimum conditions determined through multi-response optimization (*X*1 = 40 °C, *X*_2_ = 6.75 min, *X*3 = 64.04%), as quantitatively demonstrated by the OPT (fit) and OPT (exp rep.) values presented in Table 1.

For TPC, the model predicted 154.63 mg GAE/100 ml, whereas experimental validation yielded 149.77 mg GAE/100 ml, corresponding to an absolute deviation of 4.87 mg GAE/100 ml. This difference represented a relative error of 3.15% based on the predicted value (agreement ≈ 96.85%) and 3.25% relative to the experimental value (Table 1). Similarly, for FRAP, the predicted response was 13.2533 mmol TE/L, while the experimentally obtained value was 12.86 mmol TE/L, resulting in an absolute difference of 0.3933 mmol TE/L. The relative deviation was calculated as 2.97% according to the predicted value (agreement ≈ 97.03%) and 3.06% according to the experimental result (Table 1). The low percentage deviations show a high level of consistency between predicted and experimental values. This confirms the reliability of the optimization process

In conclusion, the high level of agreement between model-based optimization and experimental validation demonstrates that the proposed processing window provides not only a theoretical optimum but also a practically sustainable performance under experimental conditions (Tables 1, 2; Figure 2). The coefficient structure and the significance of the quadratic terms successfully explain the curvilinear behavior observed in the response surface plots. This suggests that the optimal parameters should not be interpreted as a single linear ‘end point', but rather as an optimal region defined by specific parameter ranges (Table 3; Figure 2). These findings demonstrate the effectiveness of thermosonication in increasing the TPC and FRAP values of broccoli juice. The optimum conditions prove the reliability and applicability of the developed model (Tables 1–3; Figure 2).

**Table 3:** Regression results for FRAP and TPC (coded quadratic models): combined coefficient table.

Response	Term	Coef	SE Coef	*t*	*P*-value	VIF
FRAP	Constant	10.8633	0.0727	149.40	0.000	
	*X*1	−0.2169	0.0912	−2.38	0.039	1.00
	*X*_2_	−1.5660	0.0912	−17.18	0.000	1.00
	*X*3	−0.5595	0.0912	−6.14	0.000	1.00
	*X*1^2^	1.4590	0.1450	10.03	0.000	1.08
	*X*_2_^2^	−0.9830	0.1450	−6.76	0.000	1.08
	*X*3^2^	−1.3040	0.1450	−8.97	0.000	1.08
	*X*1 × *X*_2_	−1.2310	0.2580	−4.77	0.001	1.00
	*X*1 × *X*3	1.2560	0.2580	4.87	0.001	1.00
	*X*_2_ × *X*3	0.3490	0.2580	1.35	0.205	1.00
TPC	Constant	139.6680	0.6440	216.99	0.000	
	*X*1	−0.9450	0.8070	−1.17	0.269	1.00
	*X*_2_	−14.6460	0.8070	−18.15	0.000	1.00
	*X*3	−8.0260	0.8070	−9.95	0.000	1.00
	*X*1^2^	−5.1100	1.2900	−3.97	0.003	1.08
	*X*_2_^2^	−30.0300	1.2900	−23.33	0.000	1.08
	*X*3^2^	−34.7300	1.2900	−26.98	0.000	1.08
	*X*1 × *X*_2_	−16.1900	2.2800	−7.09	0.000	1.00
	*X*1 × *X*3	45.6100	2.2800	19.99	0.000	1.00
	*X*_2_ × *X*3	7.5000	2.2800	3.28	0.008	1.00

Coef, regression coefficient (coded units); t, t-statistic; SE, standard error; VIF, variance inflation factor.

Terms: X1, X_2_, X3 represent coded factors; X?^2^ indicates quadratic terms; X? × X? indicates interaction terms.

### 3.2 Validation of RSM optimization using metaheuristic algorithms

**Figure 3:**
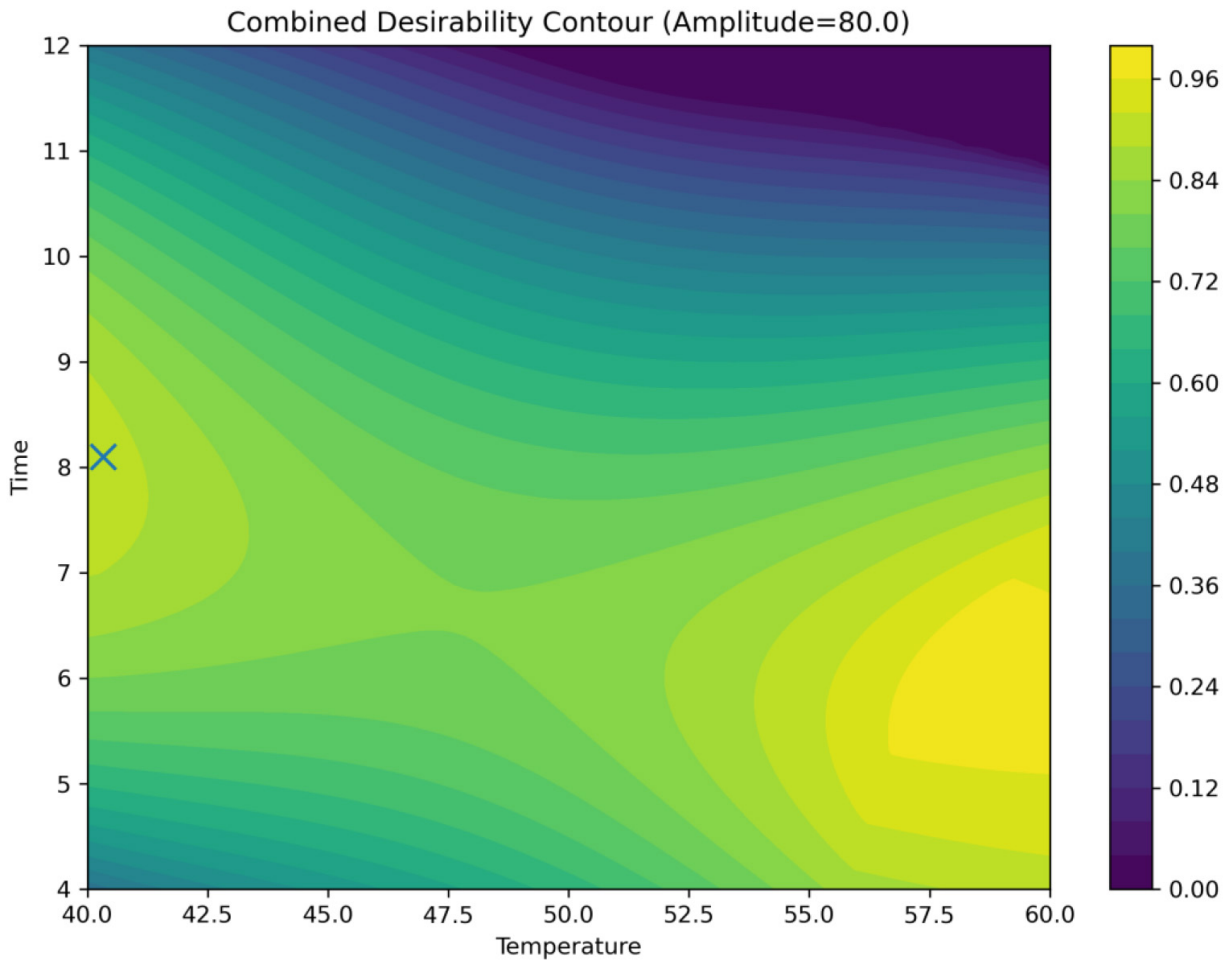
Combined desirability contour plot.

The desirability plot (Figure 3) shows how temperature and processing time affect the desirability for maximizing responses to TPC and FRAP at 80% amplitude, with values from 0 to 1 showing how this changes.

As illustrated in Figure 3, the higher-desirability areas are mostly concentrated in the lower-temperature range (around 40–45 °C) and at moderate processing times of about 6–8 min. On the other hand, increases in temperature above 55 °C and in time above 10 min decrease desirability, indicating an adverse combined effect on the response. Given the contour pattern, the relationship between temperature and time is non-linear. This aligns with the findings from the regression model, where considerable quadratic and interaction effects are significant. The desirability levels evolving slowly, with a peak rather than being sharp, imply there is an optimal region that is broad rather than a single optimum. The efficiency of this process is confirmed by its robustness, which indicates that slight changes in operating parameters in this region will not significantly affect it. The marked point on the contour indicates the result of the metaheuristic optimization algorithms (PSO/GWO), which is located in the high-desirability region. Agreement across different optimization techniques confirms that the conditions are close to optimal and that the RSM-based optimization framework is robust. It is important to recognize that this contour is a two-dimensional representation at a fixed amplitude level, and thus the global optimum obtained from full three-factor optimization may differ slightly from that manifested in this illustration.

**Figure 4:**
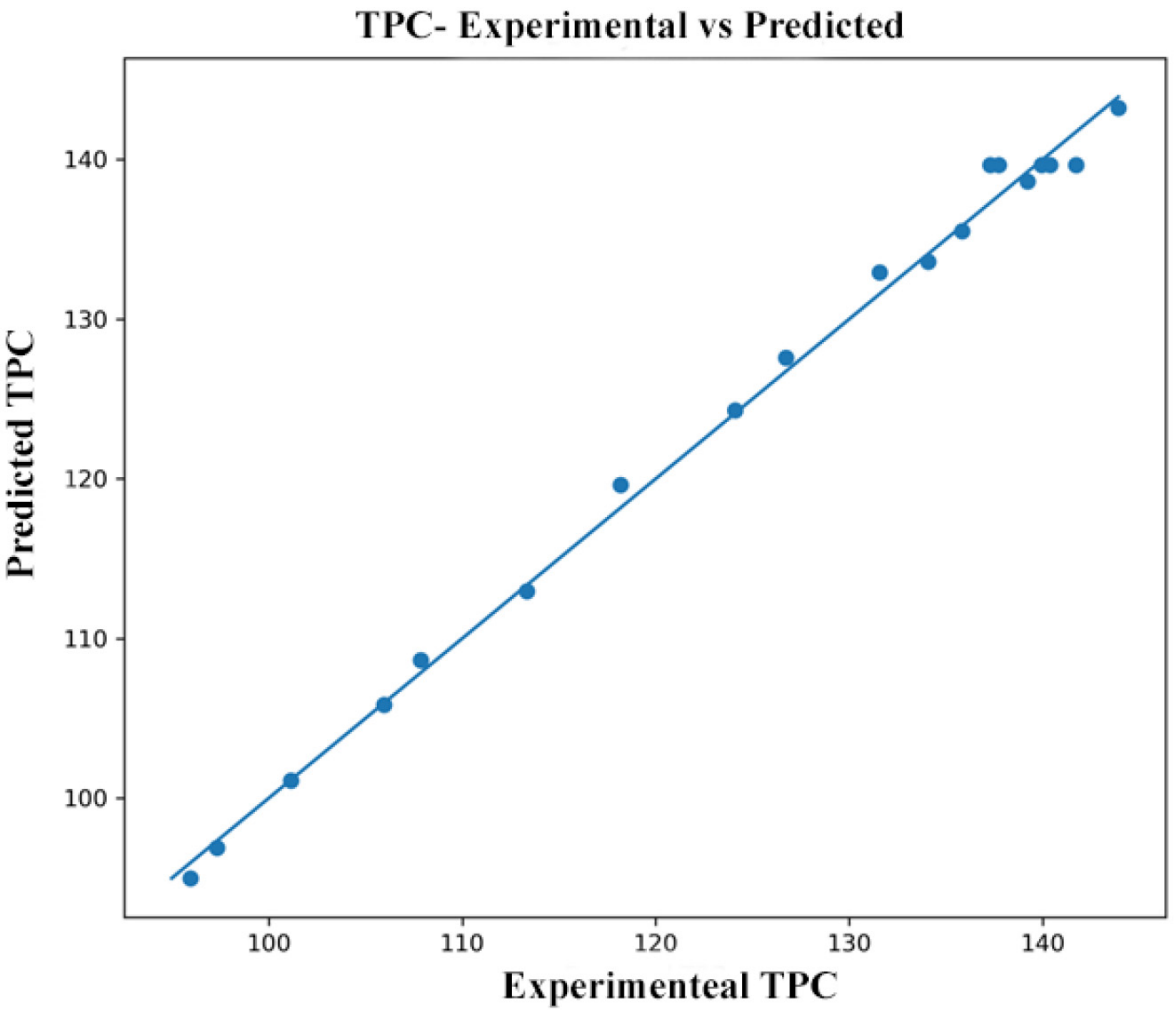
Experimental vs predicted TPC.

The experimental and GWO values for TPC are shown in Figure 4. The data points are close to the 45° line of best fit, showing excellent agreement between predicted and experimental values.

Observing the closeness of the data to the fitted regression line indicates that our quadratic model provides a fair account of the variability in TPC across the experimental design space. There are only small deviations, especially when the TPC is high. These may be due to experimental variability or slight model-approximation errors. In general, the results confirm the high predictability and robustness of the developed GWO model for TPC. According to the various model assessment stats, for instance, the high R^2^ and the non-significant lack of fit test, this observation does not pose any danger for the model.

**Figure 5:**
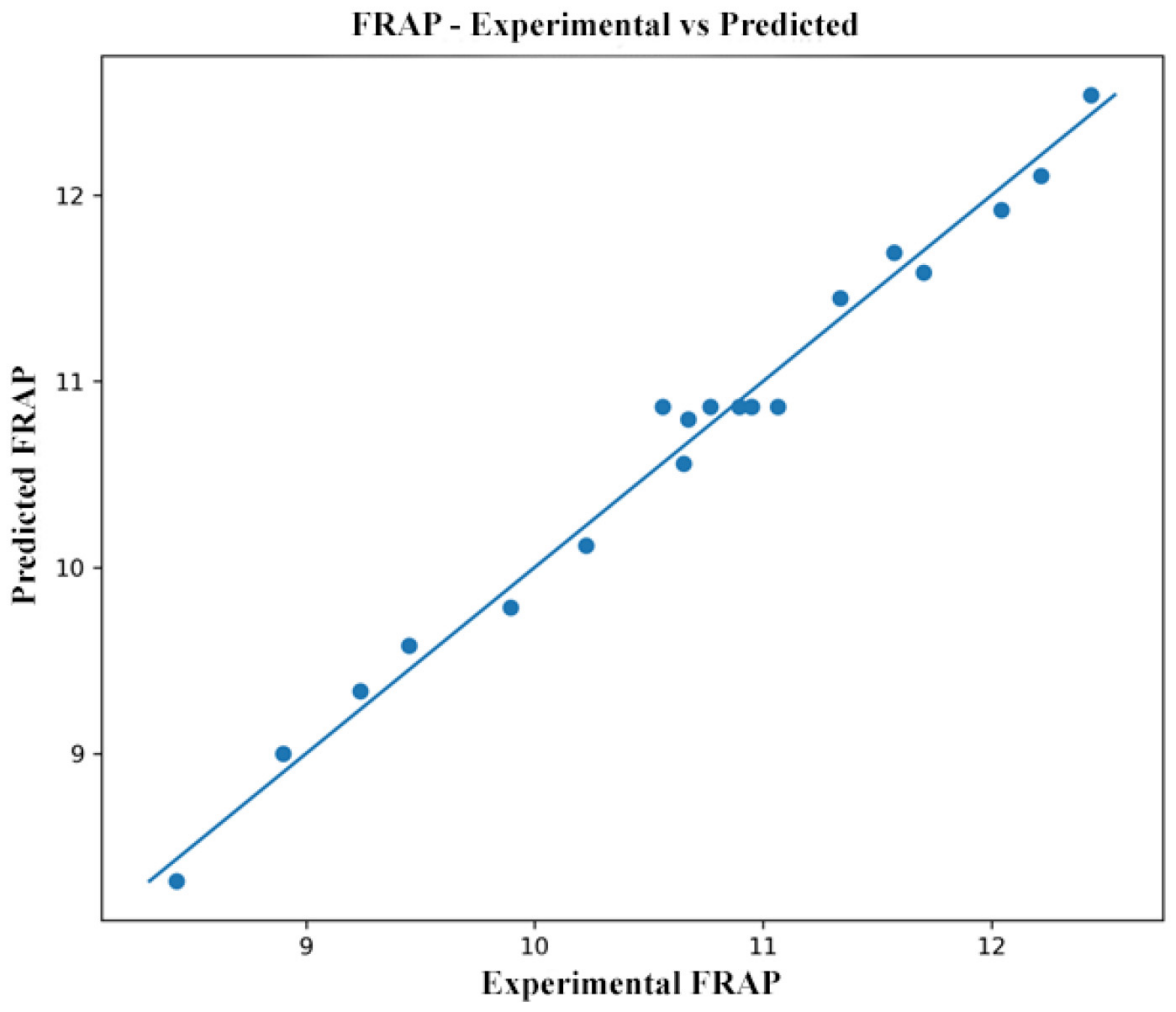
Experimental vs Predicted FRAP.

The experimental and GWO values of FRAP are shown in Figure 5. The experimental data indicates a strong correlation with the 45° reference line.

Compared to the TPC model, a slightly higher dispersion may be observed in the mid-range FRAP values. Nevertheless, the data points align with the model curve, indicating that the developed quadratic model accurately represents the antioxidant capacity. There may be small deviations here that are due to experimental variation or the complexity of the response. Most notably, there are no systematic departures from the reference line, indicating there is no bias in the model. This finding is consistent with the high *R*^2^ and the insignificant lack-of-fit, indicatingthat the model fits well and can be used to predict the FRAP.

In Table 4, the optimal conditions and corresponding response values obtained by RSM agreed with the PSO and GWO, suggesting that the optimal processing region for the simultaneous maximization of TPC and FRAP is quite robust.

**Table 4:** Comparison of optimal processing conditions obtained from RSM and metaheuristic algorithms (PSO and GWO) for simultaneous maximization of TPC and FRAP.

Method	Temperature (°C)	Time (min)	Amplitude (%)	TPC (mg GAE/100 ml)	FRAP (mmol TE/L)	Desirability
RSM (model optimum)	40.00	6.75	64.04	154.63	13.25	0.97–1.00
RSM (experimental validation)	40.00	6.75	64.04	149.77	12.86	—
PSO	40.33	8.10	69.06	153.83	13.00	1.00
GWO	40.33	8.10	69.06	153.83	13.00	1.00

RSM, response surface methodology; GWO, gray wolf optimizer; PSO, particle swarm optimization; FRAP, ferric reducing antioxidant power; TPC, total phenolic content; TE, trolox equivalents; GAE, gallic acid equivalents.

The optimization results of the metaheuristic algorithms (PSO and GWO) correlated with the RSM model results (Table 4). The predicted responses for FRAP and TPC were 13.00 mmol TE/L and 153.83 mg GAE/100 ml, respectively, with a desirability value of 1.00. There were slight differences in the combinations of angles, amplitude, and time between the maximum yield predicted by RSM and that predicted by the experimental data (40 °C, 6.75 min, 64.04% amplitude). However, the predicted response values were remarkably similar. This suggests that the optimum zone of effectiveness identified by RSM is a robust one and not merely a single-point solution. The fact that both the PSO and GWO converge on the same answer indicates we have reached the global optimum. Additionally, the metaheuristic algorithms' ability to reach a desirability value of 1.00 indicates that multiple parameter combinations can yield near-optimal responses in the design space. RSM-based optimization can be reliable and stable, and the inclusion of metaheuristic approaches further confirms the optimal processing conditions achieved, according to the findings.

### 3.3 Bioactive composition and antioxidant capacity of broccoli juice

**Figure 6:**
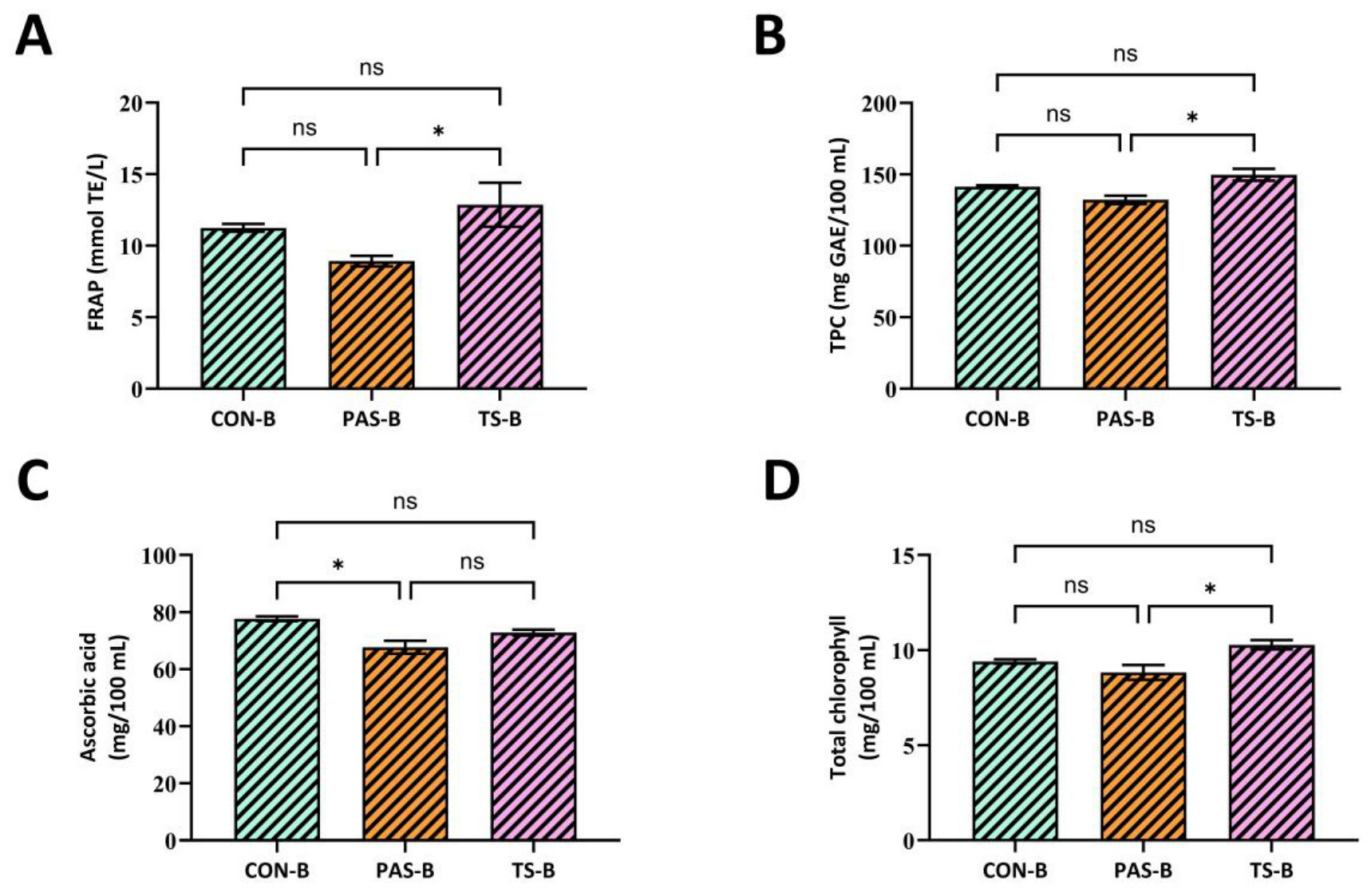
**(A)** Ferric reducing antioxidant power (FRAP), **(B)** total phenolic content (TPC), **(C)** ascorbic acid (AA), and **(D)** total chlorophyll values of broccoli juice samples subjected to different treatments: CON-B (untreated control broccoli juice), PAS-B (pasteurized broccoli juice), and TS-B (thermosonicated broccoli juice). Statistical differences among treatments were determined using one-way ANOVA. Data are presented as mean ± standard deviation (SD) (*n* = 3). n.s. (not significant), *p* ≥ 0.05; *p < 0.05.

Figure 6A shows the sensitivity of the ferric reduction antioxidant power FRAP, mmol TE/L parameter in broccoli juice samples to the type of processing applied. While the FRAP value of broccoli juice without processing (CON-B) was 11.25 mmol TE/L, the decrease to 8.93 mmol TE/L after thermal pasteurization (PAS-B) indicates that thermal processing and oxidative stress can reduce the effectiveness of components that contribute to reducing capacity, especially heat-sensitive antioxidant fractions. In the broccoli juice sample subjected to thermosonication (TS-B), the increase in FRAP to 12.86 mmol TE/L can be attributed to the relaxation of the cavitation-based matrix and increased release of antioxidant components through cellular breakdown and/or the limited overall thermal effect of the process. The results show thermosonication effectively preserves antioxidant capacity (26–28). Pairwise comparisons showeda significant difference between PAS-B and TS-B (*p* < 0.05); no such difference between CON-B and PAS-B (*p* ≥ 0.05), or between CON-B and TS-B (*p* ≥ 0.05). This finding indicates that thermosonication can significantly enhance antioxidant capacity compared to pasteurization, but the increase does not reach statistical significance relative to the control.

Figure 6B shows that theTPC varies depending on the process applied. While TPC was reported as 141.46 mg GAE/100 ml in CON-B, its decrease to 132.18 mg GAE/100 ml in PAS-B suggests that the phenolic fraction may be partially heat-sensitive. In contrast, the increase in TPC to 149.77 mg GAE/100 ml in TS-B may have resulted from thermosonication increasing cell wall permeability via cavitation-induced mechanisms. A significant difference in total phenolic content was found for PAS-B and TS-B (*p* < 0.05).

Thermosonication accelerates hydrolysis and bioactive release, and with a lower energy requirement than conventional pasteurization, it greatly preserves the sensory quality of the beverage while improving stability and safety (29).

Figure 6C shows the process-induced change in ascorbic acid (AA, mg/100 ml) content. The decrease from 77.72 mg/100 ml of AA in CON-B to 67.72 mg/100 ml in PAS-B supports the idea that degradation/oxidation processes can be accelerated during pasteurization due to the heat and oxygen-sensitive nature of ascorbic acid. The preservation of AA at 73.56 mg/100 ml in TS-B indicates a more limited loss than pasteurization and is consistent with thermosonication's ability to reduce AA loss through more controlled thermal effects. Pairwise comparisons showeda significant difference between CON-B and PAS-B (*p* < 0.05), and between PAS-B and TS-B (*p* ≥ 0.05). Nascimento et al. (30) found that thermosonication, when applied under specific conditions (3,300 W/L, 50–65 °C, 10 min), preserved the ascorbic acid (AA) content to a greater extent than conventional pasteurization (80 °C, 10 min). This result suggests that thermosonication can reduce AA loss compared to pasteurization.

Figure 6D shows that total chlorophyll levels (mg/100 ml) differ depending on the applied process. The decrease in total chlorophyll from 9.41 mg/100 ml in CON-B to 8.84 mg/100 ml in PAS-B is consistent with the susceptibility of chlorophyll to pheophytinization and oxidative transformations during heat treatment. The increase in total chlorophyll content to 10.29 mg/100 ml under TS-B treatment is consistent with the literature, which indicates that thermosonication is more effective than pasteurization (*p* < 0.05) in preserving chlorophyll stability (31, 32). This can be explained by thermosonication increasing pigment release by relaxing the cellular matrix, thereby limiting pigment losses at a lower processing level than pasteurization.

### 3.4 Phenolic compound profile of broccoli juice

Table 5 presents the concentrations of phenolic compounds in broccoli juice. The most abundant compounds were gallic acid and naringin. In the control sample (CON-B), the gallic acid content was 45.17 ± 5.56 μg/ml; after pasteurization, it decreased to 33.89 ± 0.94 μg/ml, and after thermosonication, it increased to 51.08 ± 3.10 μg/ml. Similarly, the naringin content was 3.47 ± 0.42 μg/ml in the CON-B; however, it decreased to 2.86 ± 0.08 μg/ml after pasteurization, while the highest value (4.88 ± 0.48 μg/ml) was observed in the thermosonicated samples. Among the hydroxybenzoic acids, hydroxybenzoic acid increased after thermosonication, reaching the highest value (1.08 ± 0.11 μg/ml). In contrast, vanillic acid was higher in pasteurized samples (1.02 ± 0.03 μg/ml) than in the control and thermosonicated samples. Gentisic acid was not detected in the control sample but was identified in pasteurized and thermosonicated samples at 0.45 ± 0.01 and 0.29 ± 0.03 μg/ml, respectively. Regarding flavonoids, rutin was not detected in the CON-B but was detected in the thermosonicated and pasteurized samples at 0.15 ± 0.01 and 0.27 ± 0.01 μg/ml, respectively. Neohesperidin was detected only in pasteurized samples (0.51 ± 0.01 μg/ml). In contrast, the quercetin content remained relatively stable across samples, ranging from 0.98 to 1.30 μg/ml. Similarly, hesperidin and alizarin did not show considerable variation among treatments. Conversely, no o-coumaric acid was identified in any of the samples (33).

**Table 5:** Concentrations of individual phenolic compounds in broccoli juice after different treatments (CON-B, PAS-B, and TS-B) (μg/ml).

Phenolic compounds (μg/ml)	Samples		
	CON-B	PAS-B	TS-B
Hydroxybenzoic acid	0.42 ± 0.05^a^	0.72 ± 0.02^a^	1.08 ± 0.11^b^
Gallic Acid	45.17 ± 5.56^ab^	33.89 ± 0.94^a^	51.08 ± 3.10^b^
Gentisic acid	0.00 ± 0.00^a^	0.45 ± 0.01^c^	0.29 ± 0.03^b^
Protocatechuic acid	0.07 ± 0.01^b^	0.02 ± 0.00^a^	0.02 ± 0.00^a^
Vanillic acid	0.51 ± 0.06^a^	1.02 ± 0.03^b^	0.53 ± 0.05^a^
Naringin	3.47 ± 0.42^ab^	2.86 ± 0.08^a^	4.88 ± 0.48^b^
*o*-Coumaric acid	n.d.	n.d.	n.d.
Neohesperidin	0.00 ± 0.00^a^	0.51 ± 0.01^b^	0.00 ± 0.00^a^
Coumarin	0.04 ± 0.01^b^	0.15 ± 0.01^c^	0.00 ± 0.00^a^
Rutin	0.00 ± 0.00^a^	0.27 ± 0.01^c^	0.15 ± 0.01^b^
Quercetin	1.18 ± 0.14^a^	1.30 ± 0.04^a^	0.98 ± 0.10^a^
*trans*-cinnamic acid	0.46 ± 0.06^b^	0.17 ± 0.00^a^	0.58 ± 0.06^b^
Hesperidin	0.11 ± 0.01^a^	0.11 ± 0.01^a^	0.09 ± 0.01^a^
Alizarin	0.62 ± 0.08^a^	0.56 ± 0.02^a^	0.77 ± 0.08^a^
*p*-coumaric acid	0.34 ± 0.04^a^	0.41 ± 0.01^ab^	0.54 ± 0.06^b^

PAS-B, pasteurized broccoli juice; CON-B, untreated (control) broccoli juice; TS-B, thermosonicated broccoli juice.

Significant differences among treatments are indicated by different superscript letters within the same row (p < 0.05).

Not detected (n.d.).

Studies on broccoli sprouts have revealed various phenolic compounds, including *p*-hydroxybenzoic acid, gallic acid, gallic acid hexolide derivatives, and 4-O-caffeoylquinic acid. Gallic acid, hydroxybenzoic acid, and caffeoylquinic acid derivatives are a significant portion of the phenolic profile (34). These findings support that the occurrence of gallic acid and hydroxybenzoic acid derivatives in the present study is an expected characteristic of broccoli matrices. UV treatments can promote the accumulation of certain phenolic compounds in broccoli-based products, depending on the processing conditions. Consistent with these observations, the present results showed that pasteurization and thermosonication exerted different effects on compounds such as gallic acid, hydroxybenzoic acid, vanillic acid, and naringin, suggesting that individual phenolic compounds respond differently to processing treatments. Therefore, changes in the phenolic composition of broccoli-based products should be evaluated not only in terms of total phenolic content, but also at the individual phenolic compound level.

In another study, Abd Allah et al. (9) reported that vanillic acid, pyrogallol, caffeine, catechol, and p-hydroxybenzoic acid were the major phenolic compounds present in fresh broccoli juice. Among these compounds, vanillic acid (6,415.6 μg/100 g) and pyrogallol (5,738.6 μg/100 g) were identified as the most abundant phenolics in broccoli juice. Moreover, Yikmiş et al. investigated the effects of different ultrasound and thermosonication parameters on individual phenolic compounds in broccoli juice. In that study, they demonstrated that gallic acid and naringin, which are among the major phenolic compounds in broccoli juice, were largely preserved during ultrasound and thermosonication treatments. The gallic acid concentration was reported as 49.43 μg/ml after ultrasound treatment and 49.01 μg/ml after thermosonication treatment. Similarly, the naringin content was higher under ultrasound and thermosonication conditions than under conventional pasteurization, with values of 3.67 and 4.57 μg/ml, respectively. In addition, the total amount of individual phenolic compounds was reported to be 57.04 μg/ml for ultrasound treatment and 58.14 μg/ml for thermosonication treatment. These findings suggest that conventional thermal treatments at high temperatures may degrade or structurally modify phenolic compounds, whereas emerging technologies such as ultrasound and thermosonication may better preserve them (35).

Similarly, Bas-Bellver (2024) reported that pre-treatments and drying methods applied to broccoli stem powders significantly influenced the individual phenolic profile (36). In their study, caffeic acid, gallic acid, 4-O-caffeoylquinic acid, and quercetin were identified as dominant phenolic compounds. Notably, the content of caffeic acid increased significantly in the samples treated with ultrasound, reaching a maximum of 4.92 mg/100 g in the samples that were freeze-dried with the assistance of ultrasound. This is due to the cavitation effect that occurs during ultrasonic application. This causes microchannel formation and cell wall breakdown in the plant tissue, releasing matrix-bound phenolic compounds. Previous research has shown that using ultrasound to enhance structure degradation can improve the extraction of phenolic compounds, which contribute to antioxidant properties (37).

### 3.5 Bioaccessibility

As shown in Table 6, the levels of TPC, total chlorophyll, FRAP, and AA in broccoli juice samples generally decreased as the digestion phases progressed; however, the TS-B sample maintained higher values for most parameters both before digestion and at the end of the intestinal phase. Specifically, the fact that the thermosonicated broccoli juice sample exhibited a TPC of 149.77 mg GAE/100 ml in the undigested phase and 45.61 mg GAE/100 ml in the intestinal phase, while the same parameter decreased from 132.18 mg GAE/100 ml to 34.66 mg GAE/100 ml in the PAS-B sample, suggests that thermosonication may provide better preservation than thermal pasteurization in preserving phenolic compounds. Similarly, the TPC recovery values of 30.45% for TS-B and 26.22% for PAS-B suggest that not only the initial level but also the fraction remaining accessible after digestion are strongly affected by the type of processing. This trend was also clearly reported in the study conducted on parsley juice; in that study, although the TPC value in the thermomicrowave-sonicated sample decreased from 140.77 mg GAE/100 ml to 58.18 mg GAE/100 ml at the end of the intestinal phase, this value remained above that of the pasteurized sample (44.31 mg GAE/100 ml), and the TPC recovery rate was determined as 41.34% (38). It has been reported that, in dill juice, intestinal-phase TPC levels after thermosonication remained at 34.22 ± 1.31 mg GAE/L, which was higher than that of the pasteurized sample (22.49 ± 3.07 mg GAE/L), and recovery rates favored thermosonication (39). This parallel suggests that the superiority observed in broccoli juice is not coincidental, but rather that thermosonication in green plant matrices can confer a systemic advantage that supports post-digestive phenolic stability.

**Table 6:** TPC, total chlorophyll, FRAP, and AA of broccoli juice across digestion phases and Recovery (%) under different treatments.

Parameter	Treatment	Undigested	Oral digestion	Gastric digestion	Intestinal digestion	Recovery (%)
TPC (mg GAE/100 ml)	CON-B	141.46 ± 0.69^bA^	110.33 ± 0.54^bB^	89.12 ± 0.43^bC^	40.28 ± 1.13^bD^	28.47 ± 0.78^ab^
	PAS-B	132.18 ± 2.04^cA^	102.43 ± 2.65^cB^	82.61 ± 0.64^cC^	34.66 ± 1.30^cD^	26.22 ± 0.87^b^
	TS-B	149.77 ± 2.99^aA^	116.82 ± 2.33^aB^	94.36 ± 1.89^aC^	45.61 ± 2.00^aD^	30.45 ± 1.27^a^
Total chlorophyll (mg/100 ml)	CON-B	9.41 ± 0.08^bA^	7.34 ± 0.06^abB^	5.74 ± 0.05^bC^	2.49 ± 0.27^bD^	26.41 ± 2.73^a^
	PAS-B	8.84 ± 0.28^cA^	6.71 ± 0.52^bB^	5.21 ± 0.18^cC^	2.25 ± 0.23^bD^	25.49 ± 2.23^a^
	TS-B	10.29 ± 0.18^aA^	8.02 ± 0.14^aB^	6.48 ± 0.27^aC^	3.07 ± 0.18^aD^	29.81 ± 1.60^a^
FRAP (mmol TE/L)	CON-B	11.25 ± 0.20^aA^	8.77 ± 0.15^aB^	6.52 ± 0.12^aC^	2.85 ± 0.08^aD^	25.35 ± 1.06^ab^
	PAS-B	8.93 ± 0.26^bA^	6.72 ± 0.49^bB^	5.09 ± 0.28^bC^	2.09 ± 0.05^bD^	23.42 ± 0.79^b^
	TS-B	12.86 ± 1.09^aA^	10.03 ± 0.85^aB^	7.46 ± 0.63^aC^	3.46 ± 0.47^aD^	26.81 ± 1.43^a^
AA (mg/100 ml)	CON-B	77.72 ± 0.60^aA^	60.29 ± 1.01^aB^	39.64 ± 0.31^aC^	13.11 ± 0.27^aD^	16.86 ± 0.23^a^
	PAS-B	67.72 ± 1.60^cA^	52.84 ± 1.28^cB^	34.54 ± 0.82^cC^	9.77 ± 0.41^cD^	14.44 ± 0.92^b^
	TS-B	72.84 ± 0.72^bA^	56.48 ± 1.10^bB^	37.15 ± 0.37^bC^	11.72 ± 0.59^bD^	16.09 ± 0.79^ab^

The values are shown as the mean ± standard deviation (n = 3).

Significant differences among treatments are indicated by different lowercase characters in the same column and parameter (CON-B, PAS-B, and TS-B) according to one-way ANOVA followed by Tukey's HSD test (p < 0.05).

Different uppercase letters within the same row and treatment indicate significant differences among digestion phases (Undigested, Oral, Gastric, and Intestinal) according to one-way ANOVA followed by Tukey's HSD test (p < 0.05). Uppercase letters are not shown for recovery (%).

TPC, total phenolic content; FRAP, ferric reducing antioxidant power; AA, ascorbic acid. CON-B, untreated control; PAS-B, pasteurized; TS-B, thermosonicated.

FRAP results also showed a pattern consistent with TPC findings. The antioxidant reducing capacity in broccoli juice decreased from 11.25, 8.93, and 12.86 mmol TE/L in CON-B, PAS-B, and TS-B samples, respectively, to 2.85, 2.09, and 3.46 mmol TE/L at the end of the intestinal phase; however, the highest intestinal FRAP value was still maintained in the TS-B sample. The recovery percentages of 26.81% for TS-B and 23.42% for PAS-B also support this result. The release of phenolic compounds and other antioxidants during thermosonication may account for this. In a study of vinegar made from black carrot pulp, the ultrasound-treated sample had higher FRAP values and recovery rates after digestion than the control and pasteurized samples (40). Furthermore, it has been reported that moderate sonication in Amazonian fruit juices increases the release of phenolic and carotenoids, and consequently, the DPPH, ABTS, and FRAP is enhanced (41). Therefore, the higher FRAP levels observed in broccoli juice samples, particularly in the TS-B group, are consistent with existing literature indicating that cavitation weakens cell wall integrity, thereby facilitating the release of antioxidant components from the matrix and thus increasing the biologically available fraction.

Total chlorophyll and ascorbic acid results revealed that heat- and digestion-sensitive components in broccoli juice were preserved to varying degrees, depending on the processing method. Total chlorophyll values were 9.41, 8.84, and 10.29 mg/100 ml in CON-B, PAS-B, and TS-B samples, respectively, during the undigested phase, while these values decreased to 2.49, 2.25, and 3.07 mg/100 ml at the end of the intestinal phase. The highest recovery rate of 29.81% in the TS-B sample suggests that chlorophyll derivatives are not completely broken down in the digestive medium, but are better preserved by thermosonication. The total chlorophyll level in the parsley juice sample subjected to thermomicrowave-sonication was 7.50 ± 0.33 mg/100 ml in the undigested phase and 2.35 ± 0.12 mg/100 ml in the intestinal phase, with a recovery rate of 31.30%. The pasteurized sample had a chlorophyll recovery value of 28.56% (38). Regarding ascorbic acid, although the highest initial value in broccoli juice was determined in the CON-B sample (77.72 mg/100 ml), the fact that the TS-B sample remained at a higher level of 11.72 mg/100 ml at the end of the intestinal phase than PAS-B (9.77 mg/100 ml) suggests that thermosonication causes more limited degradation of ascorbic acid compared to thermal pasteurization. Similarly, in the parsley juice study, the ascorbic acid content after thermomicrowave-sonication was found to be close to the control (123.77 mg/100 ml) at 120.58 mg/100 ml, while pasteurization reduced this value to 110.14 mg/100 ml (38). However, although ultrasound treatment significantly reduced the initial vitamin C content in the mixed tropical fruit drink, it increased the bioaccessibility value to 53.24%; The fact that pasteurization exhibited lower bioaccessibility (27.13%) shows that the initial concentration does not always directly reflect end-digestion accessibility (42). Therefore, for broccoli juice, it would be more accurate to evaluate AA not only at baseline but also by retention rates and recovery outcomes during the intestinal phase.

Overall, thermosonic treatment of broccoli juice samples emerged as the most balanced process for both preserving bioactive components at baseline levels and maintaining the accessible fraction after digestion. In contrast, pasteurization exhibited a less favorable profile, particularly with lower baseline values and more limited recovery percentages for TPC, FRAP, and total chlorophyll. As shown in previous studies, ultrasound-based treatments promote the release of phenolics, pigments, and other antioxidant components by increasing cell wall disruption in plant matrices. In contrast, excessive heat-based treatments can lead to greater losses of these components (40, 41). Furthermore, although the functional gel model enriched with anthocyanins obtained from black currant press pulp exhibited high chemical and biological antioxidant activity, the low bioaccessibility of the bioactive compounds highlighted the need for further evaluation of post-digestive preservative capability, independent of the initial composition (43). In this context, the current findings from broccoli juice indicate that thermosonication can be considered not only a preservation method but also an innovative process that supports post-digestion functional quality.

## 4 Conclusions

The present study demonstrated that thermosonication is a more efficient processing technology for improving the functional quality, antioxidant capacity, phenolic stability, and *in vitro* bioaccessibility of broccoli juice compared with conventional thermal pasteurization. The application of thermosonication significantly enhanced total phenolic content (TPC), ferric reducing antioxidant power (FRAP), total chlorophyll, and ascorbic acid retention, while also promoting higher post-digestion recovery values of bioactive compounds. In particular, thermosonicated samples maintained superior levels of TPC, FRAP, chlorophyll, and ascorbic acid throughout gastrointestinal digestion, indicating improved digestive stability and bioaccessibility of health-promoting compounds. The developed Response Surface Methodology (RSM) models successfully described the effects of processing temperature, treatment time, and ultrasound amplitude on TPC and FRAP responses, as confirmed by high determination coefficients and non-significant lack-of-fit values. Furthermore, the integration of metaheuristic optimization algorithms, namely (PSO) and (GWO), provided strong validation of the RSM-based optimization results and confirmed the robustness of the identified optimum processing region. The optimum thermosonication conditions (40 °C, 6.75 min, and 64.04% amplitude) enabled the simultaneous maximization of TPC and FRAP while preserving the phenolic composition of broccoli juice. HPLC-DAD analysis revealed that gallic acid and naringin were the pre-dominant phenolic compounds, and thermosonication preserved them better than pasteurization. These findings suggest that cavitation-induced cell disruption during thermosonication may enable the matrix-bound phenolic compounds and antioxidants to be released and stabilized. In summary, the findings show that thermosonication could be an effective alternative to traditional thermal processing for creating functional, vegetable-based beverages that are more nutritious and have greater bioactivity. In addition, the combined application of mathematical modeling and metaheuristic optimization approaches provides a powerful and reliable framework for optimizing emerging food processing technologies. Future studies may focus on sensory properties, microbial stability, shelf-life evaluation, and the *in vivo* bioaccessibility of broccoli juice processed under optimized thermosonication conditions.

## Data Availability

The raw data supporting the conclusions of this article will be made available by the authors, without undue reservation.
